# Nectin-1 and Non-muscle Myosin Heavy Chain-IIB: Major Mediators of Herpes Simplex Virus-1 Entry Into Corneal Nerves

**DOI:** 10.3389/fmicb.2022.830699

**Published:** 2022-02-28

**Authors:** Chenchen Wang, Qi Liang, Dong Sun, Yun He, Jiaxuan Jiang, Rongjie Guo, Tejsu Malla, Pedram Hamrah, Xun Liu, Zhenping Huang, Kai Hu

**Affiliations:** ^1^Department of Ophthalmology, The Affiliated Drum Tower Hospital, Medical School of Nanjing University, Nanjing, China; ^2^Department of Ophthalmology, Jinling Hospital, Medical School of Nanjing University, Nanjing, China; ^3^Tej Eye Care & Health Support Center, Kathmandu, Nepal; ^4^Tufts Medical Center, Schepens Eye Research Institute, Boston, MA, United States

**Keywords:** herpes simplex virus type 1, corneal nerve, trigeminal ganglion, Nectin-1, NMHC-IIB

## Abstract

Herpes Simplex Virus 1 (HSV-1) invades corneal nerves upon its infection of the cornea and then establishes latency in the trigeminal ganglion (TG). The latent virus in TG is often reactivated and travels back to the cornea, causing recurrent herpes simplex keratitis (HSK). The entry of HSV-1 into the corneal nerve is considered the initial step of infection resulting in HSV-1 latency and HSK recurrence. Several gD and gB receptors have been identified, including nectin-1, herpes virus entry medium (HVEM) and 3-O-sulfated heparan sulfate (3-OS-HS) as gD receptors, and non-muscle myosin heavy chain IIA (NMHC-IIA), NMHC-IIB and myelin-associated glycoprotein (MAG) as gB receptors. However, which receptors contribute to the entry of HSV-1 into corneal nerves are yet to be determined. This study observed that receptors nectin-1, HVEM, 3-OS-HS, NMHC-IIA, and NMHC-IIB, not MAG, were expressed in healthy corneal nerves. Further, we cultured TG neurons extracted from mice *in vitro* to screen for functional gD/gB receptors. Both *in vitro* siRNA knockdown and *in vivo* antibody blocking of either nectin-1 or NMHC-IIB reduced the entry and the replication of HSV-1 as shown by qPCR analysis and immunofluorescence measure, respectively. Also, we observed that the re-localization and the upregulation expression of NMHC-IIB after HSV-1 exposure were inhibited when gD receptor nectin-1 was knocked down. These data suggest that nectin-1 was the main gD receptor and NMHC-IIB was the main gB receptor in mediating HSV-1 entry and hold promise as therapeutic targets for resolving HSV-1 latency and HSK recurrence.

## Introduction

More than 90% of the population in developing countries have herpes simplex virus 1 (HSV-1) infection (Smith and Robinson, 2002; Looker et al., 2015; Harris, 2019). HSV-1 infects the cornea and causes Herpes Simplex Keratitis (HSK) (Jamali et al., 2020; Chen et al., 2021). During primary corneal HSV-1 infection, HSV-1 invades the corneal nerves, axons of the trigeminal ganglion (TG), and travels in a retrograde fashion to the neuronal cell bodies of the TG, which becomes the site for HSV-1 latency (Shukla et al., 2012; Hu et al., 2015; Yan et al., 2020). Reactivation of latent HSV-1 leads to recurrent HSK and results in visual impairment and eventual blindness (Rowe et al., 2013; Lobo et al., 2019; Yan et al., 2020). The entry of HSV-1 into the corneal nerves is essential for HSV-1 latency and recurrent HSK.

Important knowledge regarding the entry of HSV-1 into host cells has been obtained in previous studies. For a successful entry process, HSV-1 envelope glycoproteins interact with cell receptors in a specific order (Arii and Kawaguchi, 2018; Cairns et al., 2019; Madavaraju et al., 2020). Firstly, interactions of HSV-1 envelope glycoproteins gB and/or gC with heparan sulfate proteoglycans (HSPG) occur on the host cell surface (Shukla and Spear, 2001; Koujah et al., 2019). Following the initial interactions, HSV-1 envelope glycoprotein D (gD) binds to its cell receptor (gD receptor) on the host cell membrane, causing conformational changes which signal activation and recruit the fusion complex comprising of gB, gH, and gL (Hu et al., 2011; Rogalin and Heldwein, 2015; Vallbracht et al., 2021). Then gB is recognized by the gB receptor, leading to penetration and delivery of the viral nucleocapsid into the cytoplasm (Vitu et al., 2013; Musarrat et al., 2018). HSV-1 gD and gB are the main contributing glycoproteins during the entry process, which interact with their receptors on host cells, respectively, as the prerequisite for viral entry (Cairns et al., 2015).

Three central gD receptors mediating HSV-1 entry have been identified, namely nectin-1, herpes virus entry medium (HVEM), and 3-O-sulfated heparan sulfate (3-OS-HS). Nectin-1 belongs to a class of receptors in the immunoglobulin superfamily, which mediates intercellular adhesion (Samanta and Almo, 2015). HVEM is a member of the tumor necrosis factor receptor family, which is mainly expressed on the surface of immune cells to mediate signal transduction (Edwards and Longnecker, 2017). 3-OS-HS is a relatively rare modification of heparan sulfate (HS) and has currently only been known to mediate HSV-1 entry (Shukla et al., 1999; Tiwari et al., 2008; Sharthiya et al., 2017).

Cellular gB receptors include paired immunoglobulin-like type 2 receptor-α (PILRα), myelin-associated glycoprotein (MAG), non-muscle myosin heavy chain IIA (NMHC-IIA), and NMHC-IIB. PILRα is a receptor primarily expressed by immune cells but not TG (Lathe and Haas, 2017; Arii and Kawaguchi, 2018), and it is thus excluded in this study. MAG is a cell surface receptor expressed on the myelin sheath and plays an essential part in regulating axonal growth (Suenaga et al., 2010). NMHC-IIA and NMHC-IIB are heavy chain subunits of two isoforms of non-muscle myosin II proteins, which have been found to mediate HSV-1 entry in HL-60 and Vero cells (Arii et al., 2010, 2015).

**FIGURE 1:**
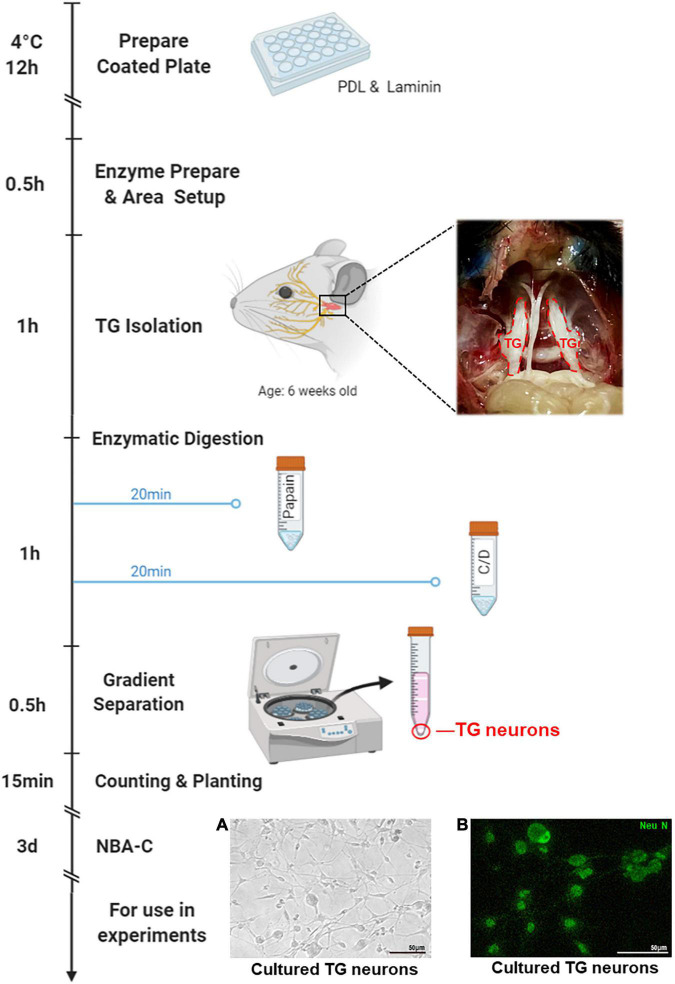
Isolation process of the TG neurons. Plate or coverslips were coated with PDL and Laminin the day before neuron isolation, followed by overnight incubation. Neuron isolation should take a total of 3.5 h. This process includes 0.5 h of setup, < 1 h to isolate TGs, < 1 h of enzymatic digestions (20 min each, with additional time for spinning down), and 1 h to separate, wash, count, and seed the neurons. The following step is > 1 h of incubation, and then 3 days of culture in Neurobasal-A Complete (NBA-C), at which point the neuron cultures are ready for later use in experiments. This figure was created with BioRender.com. **(A)** TG neurons cultured for 3 days were observed under white light. **(B)** Immunofluorescence of TG neurons cultured for 3 days stained with the neuronal marker NeuN (green).

In mouse HSK models, nectin-1 and HVEM have been reported as significant gD receptors (Karaba et al., 2011; Park et al., 2020), while contributing gB receptors have not yet been determined. It has also been reported that the deficiency of nectin-1 and HVEM receptors in mice hosts prevent HSK onset (Karaba et al., 2011). However, HSV-1 infection of the cornea involves not only corneal nerves but also corneal epithelial cells, corneal fibroblasts, corneal endothelial cells, and many other cell types. The exact receptors involved in the entry of HSV-1 into corneal nerves remain to be concluded. Furthermore, relevant receptors have not been compared against each other regarding individual contribution during the entry process. In this study, we first evaluated the distribution and the expression of previously described receptors in corneal nerves of healthy mice. In addition, since corneal nerves are the ophthalmic branch of the TG, we also verified the distribution and the expression of gD/gB receptors in the tissues of TGs. Then we isolated and cultured TG neurons from mice for *in vitro* study to explore the entry process of HSV-1 infected corneal nerve (Figure 1). Further, we screened for the functional gD/gB receptors and explored their regulatory mechanisms in cultured TG neurons *in vitro* by antibody antagonism and siRNA techniques. Additionally, we verified the role of functional receptors *in vivo* by subconjunctival injection of antibodies. Finally, we analyzed the distribution and the expression of functional gD/gB receptors in patients with HSK.

## Materials and Methods

### Cell Lines and Virus

Vero (African green monkey kidney) cells were a gift from Professor Zhiwei Wu of Nanjing University. Vero cells were cultured in Dulbecco’s modified Eagle medium (DMEM) high glucose medium (Gibco, Grand Island, NY, United States) containing 10% (v/v) fetal calf serum, 100 U/ml penicillin, and 100 g/ml streptomycin (Gibco, Grand Island, NY, United States) in a humidified incubator with 5% CO_2_ at 37°C. HSV-1 strain McKrae and HSV-1-GFP were prepared as described previously (Jamali et al., 2020; Liu et al., 2021). HSV-1 strain McKrae is a neurotoxic HSV-1 strain that causes interstitial diseases. The HSV-1 GFP is the HSV-1 strain 17 with EGFP that the EGFP protein gene replaced the UL2 gene, which is not essential for viral replication. Viruses were propagated, and titers were determined by plaque assay on Vero cells (Knipe and Spang, 1982).

### Animals

All animal experiments were conducted under the guidance of the Experimental Animal Management Committee of Jiangsu Province, China, and were approved by the Institutional Animal Care and Use Committee of Nanjing Drum Tower Hospital. C57B/6 female mice were aged 4–6 weeks were purchased from the Animal Center of Nanjing Medical University and raised in the Animal Experimental Center of Nanjing Drum Tower Hospital (Nanjing, China). The mice were kept in a pathogen-free environment with a 12:12 h light: dark cycle. Mice with no apparent abnormalities (observed under a stereomicroscope) were selected for the following experiments. All mice were anesthetized by 100 mg/kg ketamine and 20 mg/kg xylazine intraperitoneal injection. Corneas of the right eyes were scratched 5 (horizontal) × 5 (vertical) times using 30-gauge needles and inoculated with 5 μl 10^6^ PFU HSV-1 strain McKrae. Subconjunctival injection of 6 μl of specific antibody was performed before infection to investigate the role of functional receptors *in vivo*. Three days post-infection, tear samples were taken with eye swabs and added to cultured Vero cells. Virus plaque (rounding, fusion) on cultured Vero cells indicated that the mice were infected. These infected mice were then selected for follow-up experiments.

### Isolation and Culture of Trigeminal Ganglion Neurons

Plates were coated with 20 μg/ml poly-D-Lysine (Sigma-Aldrich, St. Louis, United States) and 18 μg/ml Laminin (Sigma-Aldrich, St. Louis, United States) 1 day before neuron isolation, followed by overnight incubation at 4°C. As previously described (Katzenell et al., 2017), TGs were obtained from 6 weeks old C57B/6 mice and were digested first with 40 U/ml papain (Worthington, Lakewood, NJ, United States) in HBSS at 37°C for 20 min and then with 140 U/ml collagenase type II (Worthington, Lakewood, NJ, United States) and 0.4 U/ml dispase type II (Worthington, Lakewood, NJ, United States) in HBSS at 37°C for 20 min. Gradient separation was performed at 1300 × g for 10 min in the Percoll gradient (Sigma-Aldrich, St. Louis, United States), with slow acceleration and deceleration. Next, the isolated TG Neurons were inoculated onto a well-coated plate and incubated in Neurobasal-A Complete (NBA-C): Neurobasal A medium (Gibco, Grand Island, NY, United States) supplemented with 2% B-27 Supplements (Gibco, Grand Island, NY, United States), 1% GlutaMAX (Gibco, Grand Island, NY, United States). After 3–4 days of culture, the cells were used for follow-up experiments. A diagram of the process is shown in Figure 1.

### Antibody Blocking of Major Receptors

Antibodies against nectin-1, HVEM, NMHC-IIA, and NMHC-IIB were used to block receptors on cultured TG neurons. TG neurons were first plated on a culture dish, then respective antibody was added for 2 h of incubation. Next, HSV-1 strain McKrae or HSV-1-GFP was added. Viruses were incubated at 37°C for 1 h and then replaced with the antibody-containing medium. HSV-1 entry or infection was subsequently examined. Details of the antibodies are listed in Supplementary Table 1.

### Heparinase Treatment

Heparinase selectively cleaves and degrades HS chains. TG neurons were incubated with heparinase I (1U/ml; Sigma) at 37°C for 3 h. The cells were then washed with PBS and used for HSV-1 entry assays.

### Cytotoxicity Assay

Vero cells were seeded in 96-well plates. The supernatant was then removed, and a new medium with increasing doses of heparinase I was added. After treatment for 24h, the medium was replaced with 100 μl of fresh medium containing 10 μl of Cell Counting Kit 8 (CCK-8) reagent (Dojindo, Kumamoto, Japan) for 2 h. OD values at 450 nm were measured by an MRX II microplate reader (Dynex, Chantilly, VA, United States). The cell viability values for treated cells were normalized with those of untreated cells.

### Transfection and Treatment of Cultured Trigeminal Ganglion Neurons

When the growth density of the TG neurons reached 50–70%, Lipofectamine 3000 (Invitrogen, Carlsbad, CA, United States) was used to transfect nectin-1, HVEM, NMHC-IIB or non-targeting (NC) negatively siRNA (RiboBio, Guangzhou, China) into the cells according to the manufacturer’s protocol. The cells were used for further studies 48 h after transfection. For *in vitro* study of HSV-1 infection, the virus was added to trigeminal neuron cells at 37°C and defined as zero at this point. The virus inoculum was replaced with NBA-C medium 1 h after infection. Cells were harvested at different time points for the assay. Details of siRNAs are listed in Supplementary Table 2.

### RNA Isolation and Quantitative Real-Time PCR

Total RNA was extracted from cultured cells and corneal or TG tissues using TRIzol reagent (Takara, Japan). 1 μg of total RNA was reverse transcribed into cDNA using the HiScript II Q Select RT SuperMix (Vazyme, Nanjing, China) according to the manufacturer’s instructions. Then the ChamQ Universal SYBR qPCR Kit (Vazyme, Nanjing, China) was used to perform RT-PCR on ABI QuantStudio 6 Flex (Invitrogen, Carlsbad, CA, United States). GAPDH was used as an internal control. Relative expression of genes was calculated using the 2^–ΔΔCT^ method. Primer sequences are listed in Supplementary Table 3.

### Cellular Immunofluorescence

Cultured TG neurons were inoculated on 24-well cell culture plates. After treatment, cells were fixed with 4% paraformaldehyde for 30 min. Cells were then permeabilized with 0.5% Triton X-100 in PBS for 15 min at room temperature (RT) and closed with 5% donkey serum in PBS for 1 h. Subsequently, cells were incubated with primary antibodies overnight at 4°C. After rinsing with PBST, cells were incubated with secondary antibodies for 1 h at RT. Images were obtained and analyzed using fluorescence microscopy. Primary and secondary antibodies are listed in Supplementary Table 4.

### Corneal Staining

Whole corneal staining was performed as described previously (Jamali et al., 2020). Briefly, intact corneas from mice were obtained and fixed in 4% paraformaldehyde for 30 min at RT. 0.5% Triton-X 100 was used to break the membrane for 30 min at RT. Samples were incubated in 5% bovine serum albumin for 1 h at RT to block non-specific staining. Subsequently, samples were incubated with NL557-conjugated mouse anti-β III tubulin (R&D Systems, Minneapolis, MN, United States) or FITC-anti-HSV-1 (Abcam, Cambridge, MA, United States) overnight at 4°C. Finally, sample corneas were covered with mounting media with DAPI (Abcam, Cambridge, MA, United States). Staining was observed and analyzed using the Leica Thunder system (Leica, Wetzlar, Germany). Fluorescence density was calculated using Image J and divided by the image frame area. Each image count or measurement was repeated three times and averaged. Antibodies are listed in Supplementary Table 4.

### Immunofluorescence

Mouse corneas or TG were fixed in 4% paraformaldehyde at RT overnight. The tissues were dehydrated using gradients of increasing ethanol solutions (70, 80, 90, 95, and 100%) and then embedded in paraffin. Then the tissues were serially sectioned at a thickness of 3 μm. After being baked at 65°C, they were dewaxed with xylene and hydrated with gradient ethanol. The slices were permeabilized with 0.5% Triton X-100 for 15 min. The sections were incubated for 15 min at RT and protected from light using a hydrogen peroxide blocking agent. Tissue sections were then placed in sodium citrate buffer (pH 6.0) under high pressure at 120°C for 15 min for antigen repair. 5% donkey serum was used for blocking at RT for 1 h. Then the sections were incubated with primary antibodies overnight at 4°C. After PBST rinsing, the sections were incubated with secondary antibody for 1 h at RT and covered with fixation media containing DAPI dye. The images were obtained and analyzed using the Leica Thunder system (Leica, Wetzlar, Germany). Primary and secondary antibodies are listed in Supplementary Table 4.

### Sample Collection of Patients With Herpes Simplex Keratitis

Corneal tissue samples were collected from healthy donors and recipients undergoing keratoplasty for HSK. Hematoxylin and eosin-stained sections were used for pathological diagnosis. The study was approved by Drum Tower Hospital’s Research and Ethics Committee, and all patients provided informed consent before surgery.

### Statistical Analysis

Statistical analysis was performed using GraphPad Prism 8.0. Each of the above methods included at least three biological replicates and all experiments was repeated at least three times. Differences between the groups were identified using *t*-tests. Means of three or more groups were compared using one-way ANOVA. Data are expressed as mean ± SD. Results were considered statistically significant at *P* < 0.05.

## Results

### Herpes Simplex Virus-1 gD/gB Receptors Were Expressed in the Corneal Nerves of Healthy Mice

**FIGURE 2:**
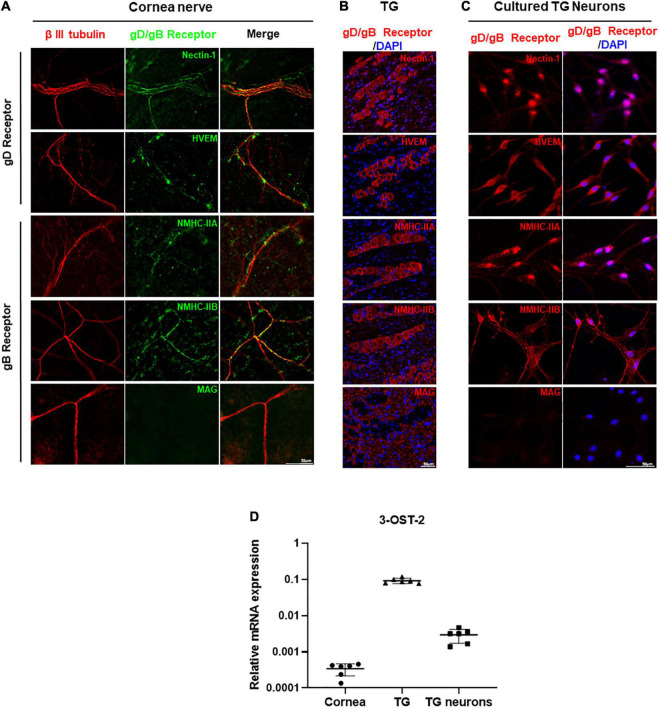
HSV-1 gD/gB receptors were expressed in the corneal nerve of healthy mice. **(A)** Immunofluorescence analysis of gD/gB receptors shows that nectin-1, HVEM, NMHC-IIA, and NMHC-IIB were expressed in corneal nerves (β III tubulin) of healthy mice, but not MAG. Shown is a single layer without z-axis stacking. **(B)** Immunofluorescence analysis of gD/gB receptors in the tissues of TGs shows that nectin-1, HVEM, NMHC-IIA, and NMHC-IIB were expressed, while MAG was only expressed in the myelin sheaths in TGs. **(C)** Immunofluorescence analysis of gD/gB receptors in the cultured TG neurons shows that nectin-1, HVEM, NMHC-IIA, and NMHC-IIB were expressed, but not MAG. **(D)** qPCR analysis of 3-OST-2 mRNA levels shows that 3-OST-2 was expressed in corneas, TGs, and cultured TG neurons from healthy mice (*n* = 6). The 3-OST-2 mRNA expression of each sample relative to GAPDH was calculated using the metric 2^–ΔCT^.

To investigate the distribution and the expression of gD/gB receptors in mouse corneal nerves, we obtained corneas from healthy mice for immunofluorescence analysis. Co-staining of nerve-specific marker β III tubulin and gD receptors in the mouse corneas showed that nectin-1 and HVEM were expressed in corneal nerves (Figure 2A). Similarly, the expression of gB receptors NMHC-IIA and NMHC-IIB were detected in corneal nerves. However, we did not find MAG in corneal nerves (Figure 2A). Since corneal nerves are the ophthalmic branch of the TG, we also verified the distribution and the expression of gD/gB receptors in the tissues of TGs. Immunofluorescence staining of gD receptors showed that nectin-1 and HVEM were expressed in TGs (Figure 2B). Immunofluorescence staining of gB receptors including NMHC-IIA, NMHC-IIB, and MAG were detected in TGs. NMHC-IIA and NMHC-IIB were mainly distributed in the cytoplasm of TG neurons (Figure 2B), while MAG was only expressed in the myelin sheaths of TGs (Figure 2B). In addition, we cultured TG neurons *in vitro* to further confirm the expression and distribution of gD/gB receptors. Immunofluorescence staining of gD receptors in cultured TG neurons showed the expression of nectin-1 and HVEM (Figure 2C). Immunofluorescence staining of gB receptors including NMHC-IIA, NMHC-IIB, and MAG in TG neurons showed that NMHC-IIA and NMHC-IIB were also detected in TG neurons (Figure 2C). However, we did not observe MAG expression in TG neurons (Figure 2C).

3-OS-HS is one of the gD receptors, and is formed by adding sulfate modification to the 3-OH position of HS by 3-O-sulfotransferase-2 (3-OST-2) in TG (Lawrence et al., 2007; Thacker et al., 2014). Since the antibody against 3-OS-HS was not available for immunofluorescence staining, we examined the expression of 3-OST-2 by qPCR analysis. The results showed that 3-OST-2 expression could be detected in corneas, TGs, as well as cultured TG neurons (Figure 2D).

### Nectin-1 of gD Receptors Played the Main Role During Herpes Simplex Virus-1 Entry Into Trigeminal Ganglion Neurons

**FIGURE 3:**
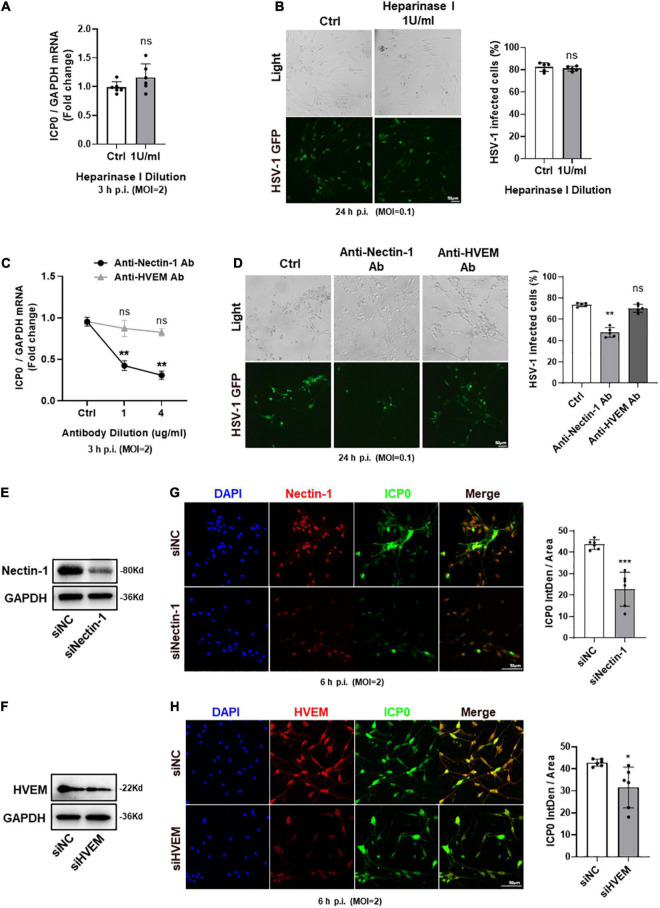
Nectin-1 of gD receptors played the main role during HSV-1 entry into TG neurons. **(A,B)** TG neurons from healthy mice cultured *in vitro* were pretreated with indicated concentrations (U/ml) of heparinase I for 3 h before infection with HSV-1 strain McKrae or HSV-1-GFP at different multiplicities of infection (MOI). Cultured TG neurons pretreated with the same concentration of DMSO were used as control (Ctrl). ICP0 mRNA levels **(A)** at 3 h post-infection (h p.i.) (*n* = 6) and the percentage of HSV-1-GFP infected cells **(B)** at 24 h p.i. (*n* = 5) were assayed. There was no difference between the heparinase I treated and the Ctrl group. **(C,D)** Cultured TG neurons were pretreated with indicated concentrations (μg/ml) of anti-nectin-1 antibody (Ab) or anti-HVEM Ab before exposure to HSV-1 strain McKrae or HSV-1-GFP at different MOIs. Cultured TG neurons with the same concentration of antibody diluent were used as Ctrl. ICP0 mRNA levels **(C)** at 3 h p.i. (*n* = 5) and the percentage of HSV-1-GFP infected cells **(D)** at 24 h p.i. (*n* = 5) were assayed. HSV-1 infection was significantly reduced in the anti-nectin-1 Ab treatment group compared to Ctrl, while there was no change in the anti-HVEM Ab treatment group. **(E,F)** Nectin-1 **(E)** or HVEM **(F)** protein levels were knocked down in cultured TG neurons transfected with targeted siRNA. **(G,H)** Cultured TG neurons infected with HSV-1 after siRNA knockdown. At 6 h p.i., immunofluorescence analysis of ICP0 expression shows that the entry of HSV-1 was inhibited in both sinectin-1 **(G)** and siHVEM **(H)** groups, but the inhibition was more significant in the sinectin-1 group. Semi-quantitative analysis of immunofluorescence was performed by Image J (*n* = 6). Bars denote SD. Ns, not significant, *P < 0.05, **P < 0.01, and ***P < 0.001 vs. Ctrl or siNC.

We used cultured TG neurons *in vitro* to further explore the entry process of HSV-1 and focused on selective gD/gB receptors. Heparinase is a glycosidic endonuclease, which has the unique ability to degrade HS present at multiple cellular sites. Heparinase I was used to eliminate HS as well as 3-OS-HS (Tiwari et al., 2006; Choudhary et al., 2011). CCK-8 assay results showed no cytotoxicity of heparinase I at the concentration of 1 U/ml reported in previous studies (Choudhary et al., 2011; Sharthiya et al., 2017) (Supplementary Figure 1). Therefore, we used heparinase I at 1 U/ml to reduce 3-OS-HS. We found no difference in ICP0 mRNA expression between heparinase-treated and untreated TG neurons (Figure 3A). We also observed no difference in the number of HSV-1-GFP-infected cells between heparinase-treated and untreated TG neurons (Figure 3B). These results suggested that 3-OS-HS may not be a crucial receptor for HSV-1 entry into TG neurons.

Next, we used neutralizing antibodies against nectin-1 and HVEM to compare the effects of their blocking upon HSV-1 infection. qPCR analysis of ICP0 mRNA expression and the number of HSV-1-GFP-infected cells were counted, respectively. We found that ICP0 mRNA was significantly decreased in the anti-nectin-1 antibody treatment group. However, there was no change in the anti-HVEM antibody treatment group (Figure 3C). The number of HSV-1-GFP-infected cells was reduced in the presence of anti-nectin-1 antibody but not anti-HVEM antibody (Figure 3D). In addition, Nectin-1 and HVEM were subjected to knockdown by their specific siRNAs and the knockdown level was confirmed by western-blot analysis (Figures 3E,F). Immunofluorescence analysis showed ICP0 protein expression was reduced after nectin-1 knockdown and the data indicated that the HSV-1 entry was blocked in the downregulation of nectin-1 expression in TG neurons (Figure 3G). Knockdown of HVEM appeared to be less effective than knockdown of nectin-1, but there was still a reduction in the expression of ICP0 protein, suggesting a role of HVEM in HSV-1 entry into TG neurons (Figure 3H). It could be concluded that nectin-1 served as a vital gD receptor to mediate HSV-1 entry into TG neurons and HVEM also potentially played a part during the entry process.

In addition to gD and gB receptors, HSPG, αvβ6-, and αvβ8-integrins also serve as HSV-1 entry receptors (Arii and Kawaguchi, 2018; Cairns et al., 2019; Madavaraju et al., 2020). HSPG has been reported to bind to gB/gC (Shukla and Spear, 2001; Koujah et al., 2019), and αvβ6- and αvβ8-integrins have been reported to bind to gH/gL (Gianni et al., 2013, 2015). To investigate whether antibody blocking or siRNA knockdown influences the expression of other HSV-1 entry receptors, we analyzed the transcriptional levels of other gD/gB receptors, HSPG, αvβ6-, and αvβ8-integrins after anti-nectin-1 antibody blocking or siRNA nectin-1 knockdown treatment. qPCR analysis presented no difference between the anti-nectin-1 antibody blocking group or the siRNA nectin-1 knockdown group and the control group (Supplementary Figure 2A).

### Non-muscle Myosin Heavy Chain-IIB of gB Receptors Mainly Mediated Herpes Simplex Virus-1 Entry Into Trigeminal Ganglion Neurons

**FIGURE 4:**
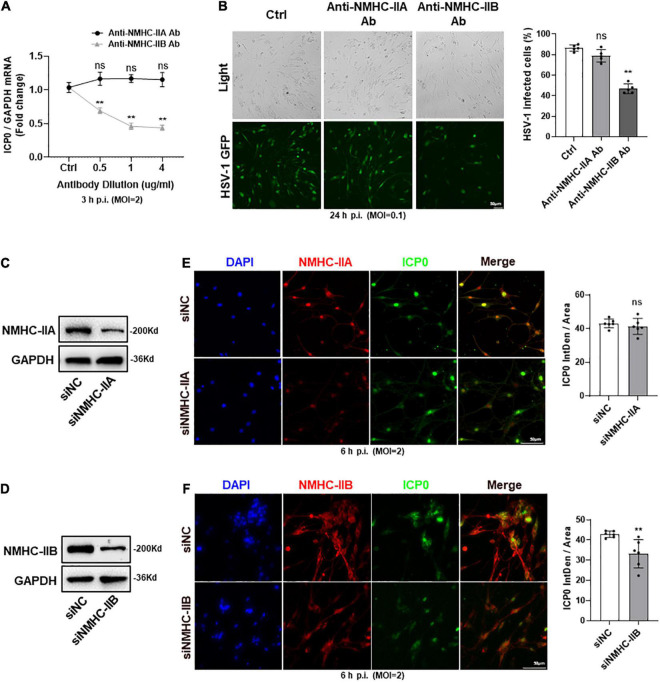
NMHC-IIB of gB receptors mainly mediated the entry of HSV-1 into TG neurons. **(A,B)** Cultured TG neurons were pretreated with anti-NMHC-IIA Ab or anti-NMHC-IIB Ab at indicated concentrations (μg/ml) before exposure to HSV-1 strain McKrae or HSV-1-GFP at different MOIs. Cultured TG neurons with the same concentration of antibody diluent were used as control (Ctrl). ICP0 mRNA levels **(A)** at 3 h p.i. (*n* = 5) and the percentage of HSV-1-GFP infected cells **(B)** at 24 h p.i. (*n* = 5) were assayed. HSV-1 infection was significantly reduced in the anti-NMHC-IIB Ab treatment group compared to Ctrl, while there was no change in the anti-NMHC-IIA Ab treatment group. **(C,D)** NMHC-IIA **(C)** or NMHC-IIB **(D)** protein levels were knocked down in cultured TG neurons transfected with targeted siRNA. **(E,F)** Cultured TG neurons infected with HSV-1 after siRNA knockdown. At 6 h p.i., immunofluorescence analysis of ICP0 expression shows that entry of HSV-1 was inhibited in the siNMHC-IIB group **(F)**, not the siNMHC-IIA group **(E)**. Semi-quantitative analysis of immunofluorescence was performed by Image J (*n* = 6). Bars denote SD. Ns, not significant, **P < 0.01 vs. Ctrl or siNC.

Inconsistency with the screening process for gD receptors, we also used antibody blocking and siRNA knockdown techniques to screen for gB receptors which played the main role in mediating HSV-1 entry into TG neurons. We found that the expression of ICP0 mRNA was decreased and the number of HSV-1-GFP infected cells was reduced by anti-NMHC-IIB antibody treatment. However, there was no change in anti-NMHC-IIA antibody treatment (Figures 4A,B). In addition, NMHC-IIA and NMHC-IIB were subjected to knockdown by their specific siRNAs and the knockdown level was confirmed by western-blot analysis (Figures 4C,D). We examined the effects of their knockdown in HSV-1 entry, and in agreement with the results of antibody blocking, immunofluorescence analysis showed that HSV-1 entry was significantly inhibited in NMHC-IIB knockdown TG neurons, while no difference was observed in NMHC-IIA knockdown TG neurons (Figures 4E,F). The results suggested that NMHC-IIB, but not NMHC-IIA, was the essential gB receptor for HSV-1 to enter TG neurons.

As mentioned above, HSPG, αvβ 6-, and αvβ8-integrins also serve as HSV-1 entry receptors (Arii and Kawaguchi, 2018; Cairns et al., 2019; Madavaraju et al., 2020). Therefore, we analyzed the transcriptional levels of other gD/gB receptors, HSPG, αvβ6- and αvβ8-integrins in the anti-NMHC-IIB antibody or siRNA against NMHC-IIB treatment group and the control group. qPCR analysis showed no difference (Supplementary Figure 2B).

### The Re-localization and Increased Expression of Non-muscle Myosin Heavy Chain-IIB in the Early Stage of Herpes Simplex Virus-1 Infection Were Inhibited by gD Receptor Nectin-1 Knockdown

**FIGURE 5:**
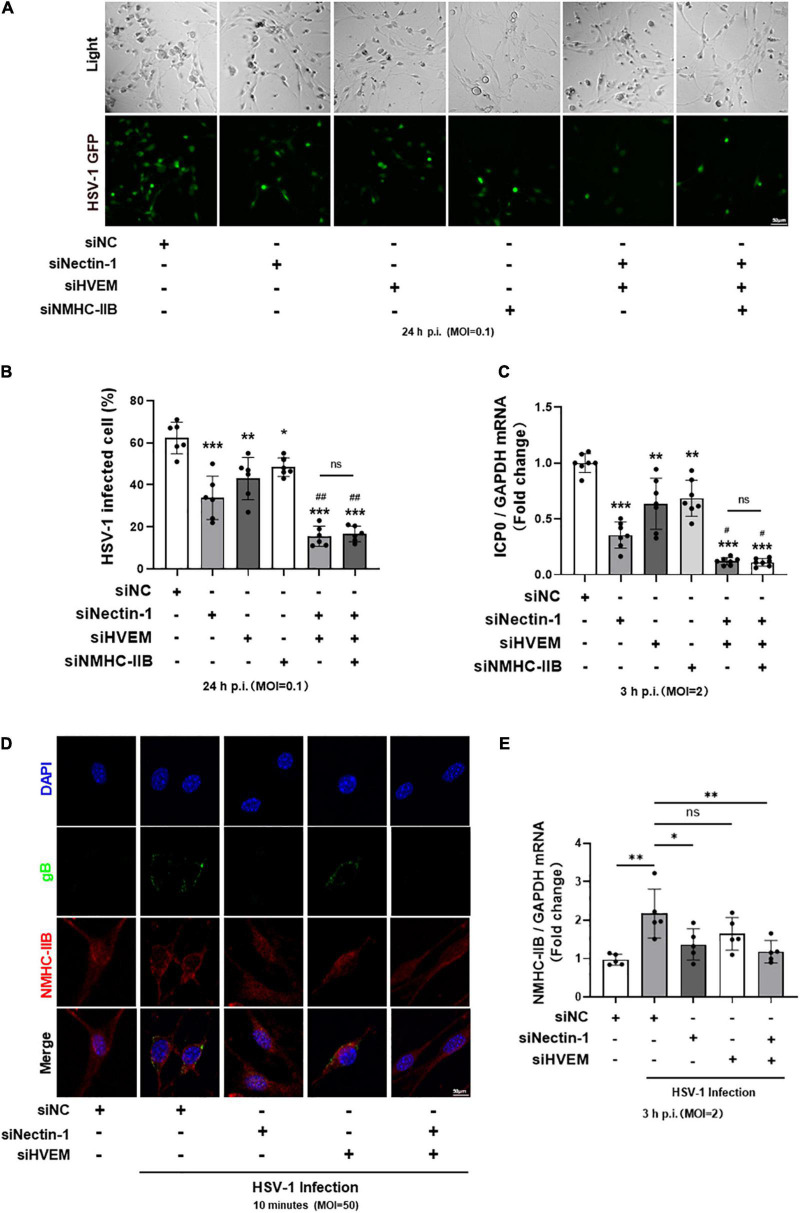
The re-localization and increased expression of NMHC-IIB in the early stage of HSV-1 infection were inhibited by gD receptor nectin-1 knockdown. **(A–C)** Cultured TG neurons were transfected with siRNA against functional HSV-1 gD/gB receptors (nectin-1, HVEM, and NMHC-IIB) alone or together. Cultured TG neurons were exposed to HSV-1 after siRNA knockdown. The percentage of HSV-1-GFP infected cells **(A,B)** at 24 h p.i. (*n* = 6) and ICP0 mRNA levels **(C)** at 3 h p.i. (*n* = 7) were assayed. Knockdown of HSV-1 functional receptors inhibited HSV-1 infection, respectively. In contrast, simultaneous knockdown of all three receptors showed no difference compared with simultaneous knockdown of two gD receptors (nectin-1 and HVEM). **(D,E)** Cultured TG neurons were transfected with siRNA against functional HSV-1 gD/gB receptors (nectin-1 and HVEM) alone or together. **(D)** Cultured TG neurons were exposed to HSV-1 strain McKrae at an MOI of 50 at 4°C for 1 h, followed by a temperature shift to 37°C for 10 min. Shown is immunofluorescence analysis of NMHC-IIB and gB expression and localization. HSV-1 infection increased the distribution of NMHC-IIB on the cell surface, while nectin-1 knockdown or nectin-1/HVEM co-knockdown prevented this alteration. **(E)** Cultured TG neurons were exposed to HSV-1 and NMHC-IIB mRNA levels at 3 h p.i. (*n* = 5) were assayed. HSV-1 infection upregulated the NMHC-IIB mRNA levels, while nectin-1 knockdown or nectin-1/HVEM co-knockdown prevented this upregulation. Bars denote SD. Ns, not significant, *P < 0.05, **P < 0.01, and ***P < 0.001 vs. Ctrl or siNC. ^#^*P* < 0.05, ^##^*P* < 0.01 vs. sinectin-1 pretreatment group.

To further explore the relationship between gD receptors nectin-1/HVEM and gB receptor NMHC-IIB in HSV-1 entry into TG neurons, we transfected siRNAs of the three receptors alone or together to compare their effects during the process. Downregulation of the three receptors alone all significantly inhibited HSV-1 entry into TG neurons (Figures 5A–C). In addition, an obvious reduction of HSV-1 entry was obtained when nectin-1 and HVEM siRNAs were co-transfected into TG neurons (Figures 5A–C), suggesting that nectin-1 and HVEM played an important part in HSV-1 entry. However, when gD receptors nectin-1/HVEM and gB receptor NMHC-IIB were simultaneously downregulated, the combined inhibitory effect of nectin-1/HVEM/NMHC-IIB knockdown in HSV-1 entry into TG neurons was the same as nectin-1/HVEM co-knockdown (Figures 5A–C).

As shown in this study and reported by previous studies, small amounts of NMHC-IIB were detectable on the surface of mock-infected cells (Figures 2A–C) (Vicente-Manzanares et al., 2009). NMHC-IIB is upregulated on the cell surface after HSV-1 exposure, and the re-localization of NMHC-IIB during the initiation of viral entry might be required for efficient viral entry (Arii et al., 2010, 2015; Orgaz et al., 2020). Therefore, we wondered whether the results showed in Figures 5A–C might be related to the re-localization of NMHC-IIB. We detected the expression and the localization of NMHC-IIB in the early stage of HSV-1 infection in the absence of gD receptor nectin-1/HVEM. Immunofluorescence staining of gB and NMHC-IIB showed the upregulated expression of NMHC-IIB and the co-localization of NMHC-IIB with gB on the cell surface within 10 min post-infection in TG neurons (Figure 5D). Surprisingly, the upregulation and the co-localization were inhibited when nectin-1 was knocked down or nectin-1/HVEM were co-knocked down (Figure 5D). Similarly, the qPCR analysis showed that the expression of NMHC-IIB mRNA was increased after HSV-1 infection in TG neurons and this was inhibited when nectin-1 was knocked down or nectin-1/HVEM were co-knocked down (Figure 5E). However, knockdown of HVEM individually appeared to be less effective in the re-localization of NMHC-IIB and its co-localization with gB compared with nectin-1 knockdown or nectin-1/HVEM co-knockdown (Figure 5D). Also, there was no change in the expression of NMHC-IIB mRNA when HVEM was knocked down (Figure 5E). In addition, as shown in Figure 4, NMHC-IIA may not be a gB receptor for HSV-1 entry into TG neurons. We further observed that there was no difference in the localization of NMHC-IIB after HSV-1 exposure when NMHC-IIA was knocked down (Supplementary Figure 3). These results may indicate that the upregulation of NMHC-IIB on the cell surface and its increased expression in the early stage of HSV-1 infection were inhibited in the absence of gD receptors, especially nectin-1.

### Subconjunctival Injection of Anti-Nectin-1/Anti-Non-muscle Myosin Heavy Chain-IIB Antibody *in vivo* Inhibited Herpes Simplex Virus-1 Entry Into Corneal Nerves

**FIGURE 6:**
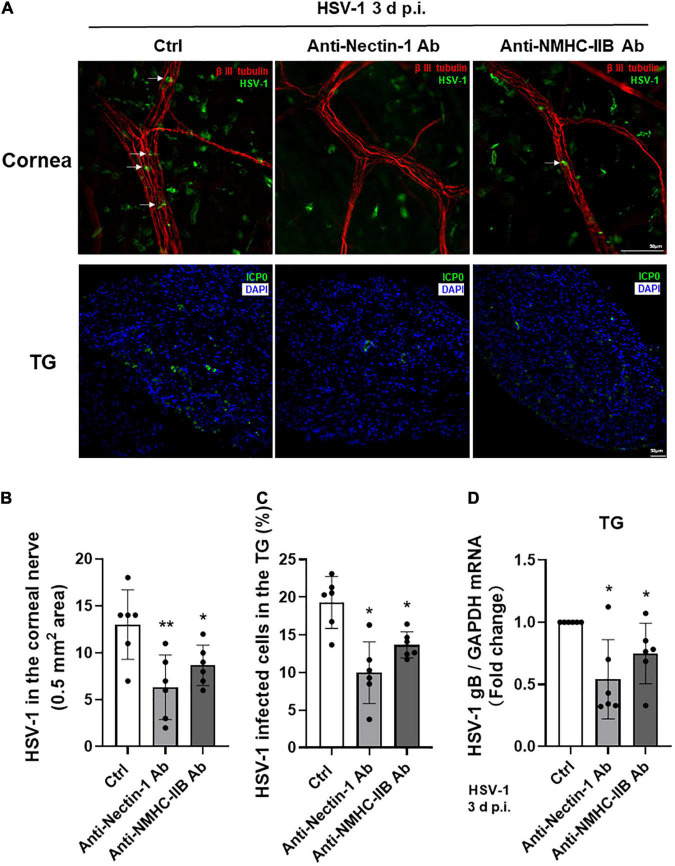
Subconjunctival injection of anti-nectin-1/anti-NMHC-IIB antibody *in vivo* inhibited HSV-1 entry into corneal nerves. **(A–D)** Antibodies of functional receptors were injected subconjunctivally in mice before HSV-1 infection of the cornea. Cornea and TG samples were harvested at 3 d p.i. The localization of HSV-1 **(A)** and the percentage of infected cells **(B–C)** were shown in corneas and TGs (*n* = 6). HSV-1 gB transcription levels **(D)** in TG were assayed. HSV-1 infection of cornea nerves and TGs were inhibited in both antibody treatment groups (*n* = 6). Shown is a single layer without z-axis stacking. Bars denote SD. *P < 0.05, **P < 0.01 vs. Ctrl.

Based on the above results, we concluded that gD receptor nectin-1 and gB receptor NMHC-IIB might together mediate HSV-1 entry into cornea nerves. Therefore, we performed subconjunctival injection of anti-nectin-1 or anti-NMHC-IIB antibodies in mice before HSV-1 infection of the cornea to explore the role of nectin-1 or NMHC-IIB *in vivo*. Mice corneas and TGs were collected at 3 days post-infection for analysis. Immunofluorescence staining of the corneas showed that the number of HSV-1 entering corneal nerves was significantly reduced in both anti-nectin-1 and anti-NMHC-IIB antibody treatment groups compared with the control group, but there was no difference between the two treated groups (Figures 6A,B). Immunofluorescence and qPCR analysis of TG samples also showed a significant reduction of HSV-1 entry in anti-nectin-1 and anti-NMHC-IIB antibody treatment groups (Figures 6A,C,D). The results indicated that both anti-nectin-1 and anti-NMHC-IIB antibodies could reduce HSV-1 latency in TG by inhibiting HSV-1 entry into TG neurons.

### Nectin-1 and Non-muscle Myosin Heavy Chain-IIB Were Upregulated in the Corneas of Patients With Herpes Simplex Keratitis

**FIGURE 7:**
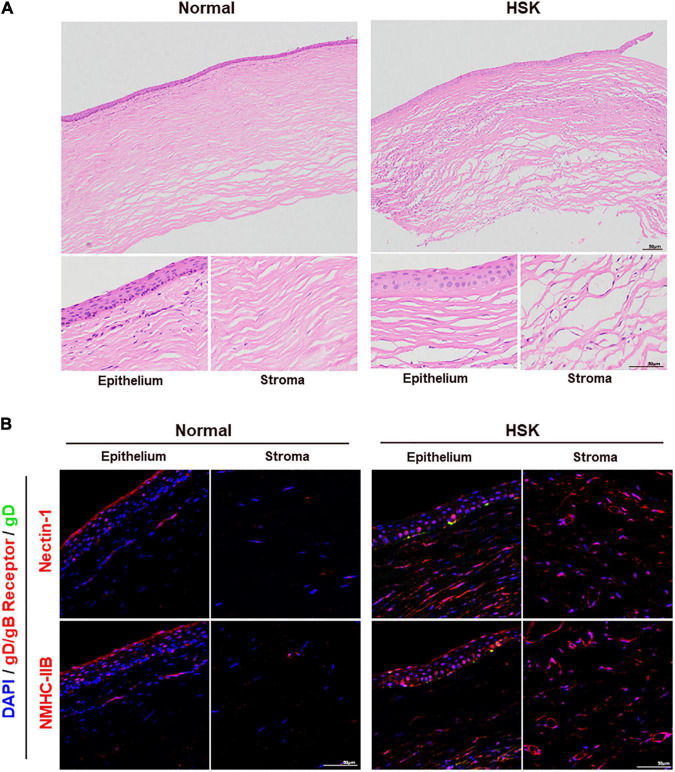
Nectin-1 and NMHC-IIB were upregulated in the corneas of patients with HSK. **(A)** Shown is HE staining of corneal sections from healthy donors and patients with HSK (*n* = 3). Corneas of patients with HSK presented significant corneal thickening with extensive infiltration of inflammatory cells and vascularization. **(B)** Shown is immunofluorescence analysis of HSV-1 gD, nectin-1, and NMHC-IIB expression and localization in corneal sections of healthy donors and patients with HSK (*n* = 3). The expression of nectin-1 and NMHC-IIB was significantly increased throughout the cornea.

Corneal section samples were collected from both healthy donors and patients with HSK for analysis. HE staining revealed corneal thickening, stromal thinning, massive inflammatory cell infiltration, loss of normal orderly structure, and neovascularization in corneal samples of patients with HSK (Figure 7A). Immunofluorescence staining of gD and functional receptors nectin-1/NMHC-IIB showed that nectin-1 and NMHC-IIB were expressed in corneal epithelium and stroma in samples of healthy donors. Upregulation of nectin-1 and NMHC-IIB were observed in corneal samples of patients with HSK, especially in HSV-1 infected areas (Figure 7B).

## Discussion

**FIGURE 8:**
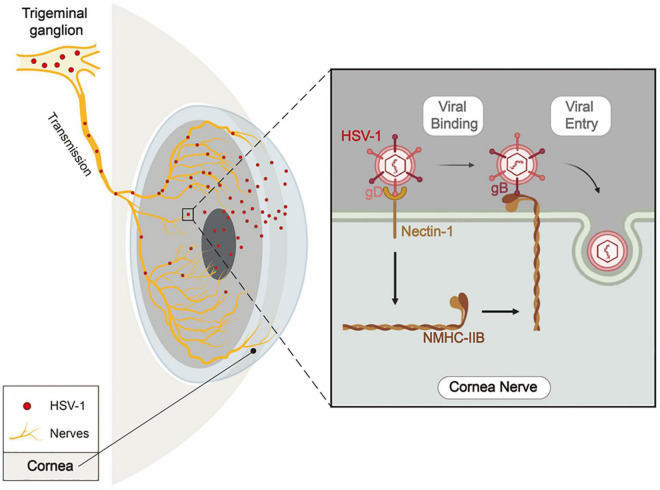
Schematic diagram of nectin-1 and NMHC-IIB mediating HSV-1 entry into corneal nerves. When infecting the cornea, HSV-1 enters corneal nerves, travels along TG’s ophthalmic branches, and finally establishes latency in TG. During the entry of HSV-1 into corneal nerves, HSV-1 gD binds to nectin-1 as the first step. This results in the attachment of the virus to the cell surface and the upregulated expression of NMHC-IIB on the cell surface. Then, HSV-1 gB binds to NMHC-IIB, allowing the virus to fuse with the cell and thus completes its entry process.

The entry of HSV-1 into TG via corneal nerves is essential for HSV-1 latency and HSK recurrence, but little is known about the underlying molecular mechanisms. Here we conducted a systematic study to research this problem. First, we evaluated the expression of previously reported HSV-1 gD/gB receptors in corneal nerves of healthy mice. Then we selected receptors with the high expression levels — nectin-1, HVEM, 3-OS-HS, NMHC-IIA, and NMHC-IIB, and investigated the effects of their blocking and knockdown in HSV-1 entry in cultured TG neurons and mouse models, respectively. Finally, we identified nectin-1 as the main gD receptor and NMHC-IIB as the main gB receptor in mediating HSV-1 entry into corneal nerves (Figure 8).

Previous studies have reported the expression of HSV-1 gD/gB receptors nectin-1, HVEM, MAG, and NMHC-IIA in TG, but not PILRα (Lathe and Haas, 2017). Our study comprehensively examined the expression of HSV-1 entry receptors in corneal nerves, TG, and cultured TG neurons of healthy mice. Our results demonstrated the high expression levels of nectin-1, HVEM, NMHC-IIA, and NMHC-IIB, while the expression of MAG was undetected either in corneal nerves or cultured TG neurons (Figures 2A–C). The results could be attributed to the fact that ophthalmic branches of the TG nerves lose their glial cell wrapping after penetrating through corneal stroma into the epithelium (Yang et al., 2018), and glial cell wrapping was found absent in cultured neuronal cells *in vitro* (Katzenell et al., 2017). The results of our study also presented different distribution patterns of the gD/gB receptors, as shown in Figure 2A. Nectin-1 and NMHC-IIB showed a smooth, diffusive distribution along the cell membrane, whereas HVEM and NMHC-IIA showed a dotted distribution. These results imply that HVEM and NMHC-IIA might accumulate at specific locations on corneal nerves. Whether this phenomenon is related to HSV-1 entry requires further investigation.

In this study, the expression of 3-OS-HS was represented by 3-OST-2, and the expression of 3-OST-2 was confirmed in the cornea, TG, and cultured TG neurons of healthy mice. The expression of 3-OS-HS was not directly confirmed in the mouse corneal nerves and is considered a limitation of this study. Heparinase I is used to eliminate HS as well as 3-OS-HS (Tiwari et al., 2006; Choudhary et al., 2011), and heparinase I treatment in our research showed that 3-OS-HS might not be essential for HSV-1 to enter cultured TG neurons (Figures 3A,B). However, a previous study has reported that 3-OS-HS mediate HSV-1 infection in dorsal root ganglia explant model and single-cell neurons. Therefore, the role of 3-OS-HS in HSV-1 entry into the corneal nerves needs to be explored in additional research.

We found that nectin-1 served as a vital role of the gD receptor to mediate the entry of HSV-1 into cornea nerves both *in vitro* (Figure 3) and *in vivo* (Figure 6). Previous studies have shown that the deficiency of nectin-1 and HVEM receptors in mice hosts prevented HSK onset (Karaba et al., 2011). Nectin-1 has been reported as the primary receptor for HSV-1 in various tissues, especially the nerve tissue (Kopp et al., 2009; Samanta and Almo, 2015). Christine Wilcox’s study showed that soluble recombinant nectin-1 or antibodies of nectin-1 both inhibit HSV-1 entry into cultured dorsal root ganglion neurons *in vitro (Richart et al., 2003)*. The similarity with our study is that we both found that antibody blocking of nectin-1 could inhibit HSV-1 entry into neurons. However, the main ideas of studies were different in that they were concerned about the role of nectin-1 in the dorsal root ganglion (DRG), while we focused on the role of nectin-1 in the corneal nerves and TG. Although both DRG and TG are primary sensory neurons, they have distinct embryonic origins (Megat et al., 2019), and there are differences in their cellular populations (Price and Flores, 2007). In addition, this study further used siRNA against nectin-1 *in vitro* and explored the role of nectin-1 in corneal nerves *in vivo*. The present study provides direct evidence supporting the role of nectin-1 in mediating HSV-1 entry into the corneal nerve.

Here, we found that co-knockdown of nectin-1 and HVEM inhibits HSV-1 entry by nearly 80% and is more effective than knockdown of nectin-1/HVEM alone (Figures 5A–C). The results suggest that HVEM might act as a functional receptor during HSV-1 entry into corneal nerves. Notably, HVEM expression is relatively lower in TGs and cultured TG neurons and is overexpressed in TGs only after HSV-1 infection (Kovacs et al., 2009; Karaba et al., 2012). Upregulated HVEM in the cornea has been associated with local immune regulation and viral reactivation (Edwards et al., 2017; Venuti et al., 2019; Tormanen et al., 2020). The results indicate that HVEM might play a more significant role in the later stage of HSV-1 infection than in its entry process, which also remains to be further elucidated.

Earlier studies have also described the role of non-muscle myosin II in viral transport and export during the viral life cycle of HSV-1 (Antoine and Shukla, 2014; Tan et al., 2019). Our study complemented the roles of NMHC-IIA and NMHC-IIB in mediating HSV-1 entry into corneal nerves. Our results showed that NMHC-IIB was the primary receptor for HSV-1 entry, while NMHC-IIA appeared less relevant. However, sufficient evidence requires studies of a wider variety of cell types and a broader range of HSV-1-associated diseases. The underlying mechanisms of virus binding sites, conformation, and specific mediation of virus entry remain obscure. Further investigation is much needed to provide substantive supporting facts.

Here, we observed that the combined inhibitory effect of nectin-1/HVEM/NMHC-IIB knockdown on HSV-1 entry was the same as nectin-1/HVEM co-knockdown (Figures 5A–C). It might be attributed to the results that the re-localization and the increased expression of NMHC-IIB were inhibited when gD receptors nectin-1/HVEM were co-knocked down (Figures 5D,E). Previous studies have found that the binding of gD to nectin-1 promotes the release of Ca^2+^ from the cell membrane (Cheshenko et al., 2007; Saurav et al., 2021). Ca^2+^ is an essential co-factor in the activation of myosin light chain kinase (MLCK), which controls the function and localization of NM-II through phosphorylation (Martinsen et al., 2014). In addition, the initial binding of gD to cellular receptors causes conformational changes in gB through the gH/gL complex (Eisenberg et al., 2012). These results might suggest that the binding of gD to gD receptors regulated both the virus and the infected cells to allow more efficient entry of the virus.

We also found that the re-localization of NMHC-IIB was accompanied by the increased distribution of gB on the cell surface (Figure 5D). The re-localization of NMHC-IIB may be more favorable for gB binding and HSV-1 entry, and it may lead to more damage of the corneal nerves and increase latent virus in TG. However, more direct data are needed to support this conclusion. In addition, we observed the increased expression of NMHC-IIB in TG neurons after HSV-1 exposure. It has been reported that members of the myosin family are involved in multiple steps of HSV-1 replication (van Leeuwen et al., 2002; Roberts and Baines, 2010) and this may explain the increased expression of NMHC-IIB upon HSV-1 infection. Further clarification of the precise roles of myosin in HSV-1 infection may elucidate the molecular basis of the multiple aspects.

In HSK patients, we observed the upregulated expression of nectin-1 and NMHC-IIB in both corneal epithelium and stroma (Figure 7B). The upregulated expression of nectin-1 and NMHC-IIB in corneal epithelium was mainly observed in HSV-1-infected areas, suggesting that HSV-1 might promote its cell-to-cell spread by increasing the expression of nectin-1 and NMHC-IIB. In contrast, the increased expression of nectin-1 and NMHC-IIB in the corneal stroma was widespread. The results are likely associated with scar formation, inflammatory cell infiltration, and vascular neogenesis, and it should be taken into consideration that the cells involved in these pathological processes, such as fibroblasts and inflammatory cells, also express nectin-1 and NMHC-IIB (Ma and Adelstein, 2014; Samanta and Almo, 2015). It is necessary to distinguish these cells to elucidate the mechanism of this upregulation.

The clarification of the mechanisms regarding HSV-1 entry into corneal nerves is of extreme clinical importance. The present study fills this research gap and holds practical implications for the future prevention of HSV-1 latency and recurrent HSK. Our results also point out that the development of novel HSK treatments to prevent HSV-1 latency should focus on the two gD receptors (nectin-1 and HVEM). Though NMHC-IIB also appeared to play an important role in HSV-1 entry, knockdown of NMHC-IIB only presented partial inhibition, which implied the possible existence of other gB receptors. In addition, a unified theory and general model of HSV-1 entry deserves further inquiry and exploration. As HSV-1 infection of corneal nerves remains a significant cause of morbidity, this study provides important insights to guide future research work.

## Data Availability Statement

The raw data supporting the conclusions of this article will be made available by the authors, without undue reservation.

## Ethics Statement

The studies involving human participants were reviewed and approved by Research and Ethics Committee, Nanjing Drum Tower Hospital, The Affiliated Hospital of Nanjing University Medical School. The patients/participants provided their written informed consent to participate in this study. The animal study was reviewed and approved by Ethics Committee on Laboratory Animals, Nanjing Drum Tower Hospital, The Affiliated Hospital of Nanjing University Medical School.

## Author Contributions

CW and KH designed the project. CW, QL, and KH prepared the manuscript. CW performed most of the experiments *in vitro*. QL, OS, and YH performed most of the experiments *in vivo*. CW, QL, YH, and RG prepared HSV-1 and cultured TG neurons. CW and QL analyzed the data. JJ, TM, PH, and KH reviewed and revised the manuscript. XL, ZH, PH, and KH supervised the study. KH financially supported the study. All authors contributed to the article and approved the submitted version.

## Conflict of Interest

The authors declare that the research was conducted in the absence of any commercial or financial relationships that could be construed as a potential conflict of interest.

## Publisher’s Note

All claims expressed in this article are solely those of the authors and do not necessarily represent those of their affiliated organizations, or those of the publisher, the editors and the reviewers. Any product that may be evaluated in this article, or claim that may be made by its manufacturer, is not guaranteed or endorsed by the publisher.

## Supplementary Material

Supplementary Figure 1 No cytotoxicity of heparinase I at 1U/ml. Vero cells were incubated in a medium with increasing doses of heparinase I for 24 h. CCK-8 assay results showed that there was no cytotoxicity of heparinase I at 1U/ml.

Supplementary Figure 2 Antibodies and siRNA of nectin-1 and NMHC-IIB did not affect other receptors involved in HSV-1 entry. **(A)** Cultured TG neurons were treated with antibody/siRNA of nectin-1 (*n* = 3). Cultured TG neurons with antibody diluent or NC siRNA were used as Ctrl or siNC. qPCR analysis showed that gD/gB receptors except nectin-1, HSPG, αvβ6- and αvβ8-integrins mRNA levels were no changes in the antibody/siRNA treatment groups compared to Ctrl or siNC. **(B)** Cultured TG neurons were treated with antibody/siRNA of NMHC-IIB (*n* = 3). Cultured TG neurons with antibody diluent or NC siRNA were used as Ctrl or siNC. qPCR analysis showed that gD/gB receptors except NMHC-IIB, HSPG, αvβ6- and αvβ8-integrins mRNA levels were no changes in the antibody/siRNA treatment groups compared to Ctrl or siNC. Bars denote SD. ns: not significant, ^***^*P* < 0.001 vs. Ctrl or siNC.

Supplementary Figure 3 No difference in the localization of NMHC-IIB after HSV-1 exposure when NMHC-IIA was knocked down. Cultured TG neurons were exposed to HSV-1 strain McKrae at an MOI of 50 at 4°C for 1 h, followed by a temperature shift to 37°C for 10 min. Shown is immunofluorescence analysis of NMHC-IIB and gB expression and localization. There was no difference in the localization of NMHC-IIB after HSV-1 exposure when NMHC-IIA was knocked down.
