# Supplementary figures and images for: Nectin-1 and Non-muscle Myosin Heavy Chain-IIB: Major Mediators of Herpes Simplex Virus-1 Entry Into Corneal Nerves
Source: Front Microbiol. 2022 Feb 28;13:830699. doi: 10.3389/fmicb.2022.830699 (PMC8919962; doi:10.3389/fmicb.2022.830699)

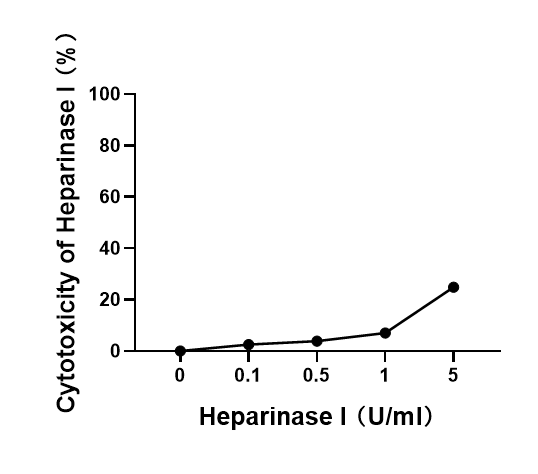

Supplement: Supplementary Figure 1 — No cytotoxicity of heparinase I at 1U/ml. Vero cells were incubated in a medium with increasing doses of heparinase I for 24 h. CCK-8 assay results showed that there was no cytotoxicity of heparinase I at 1U/ml. [file Image_1.TIF]

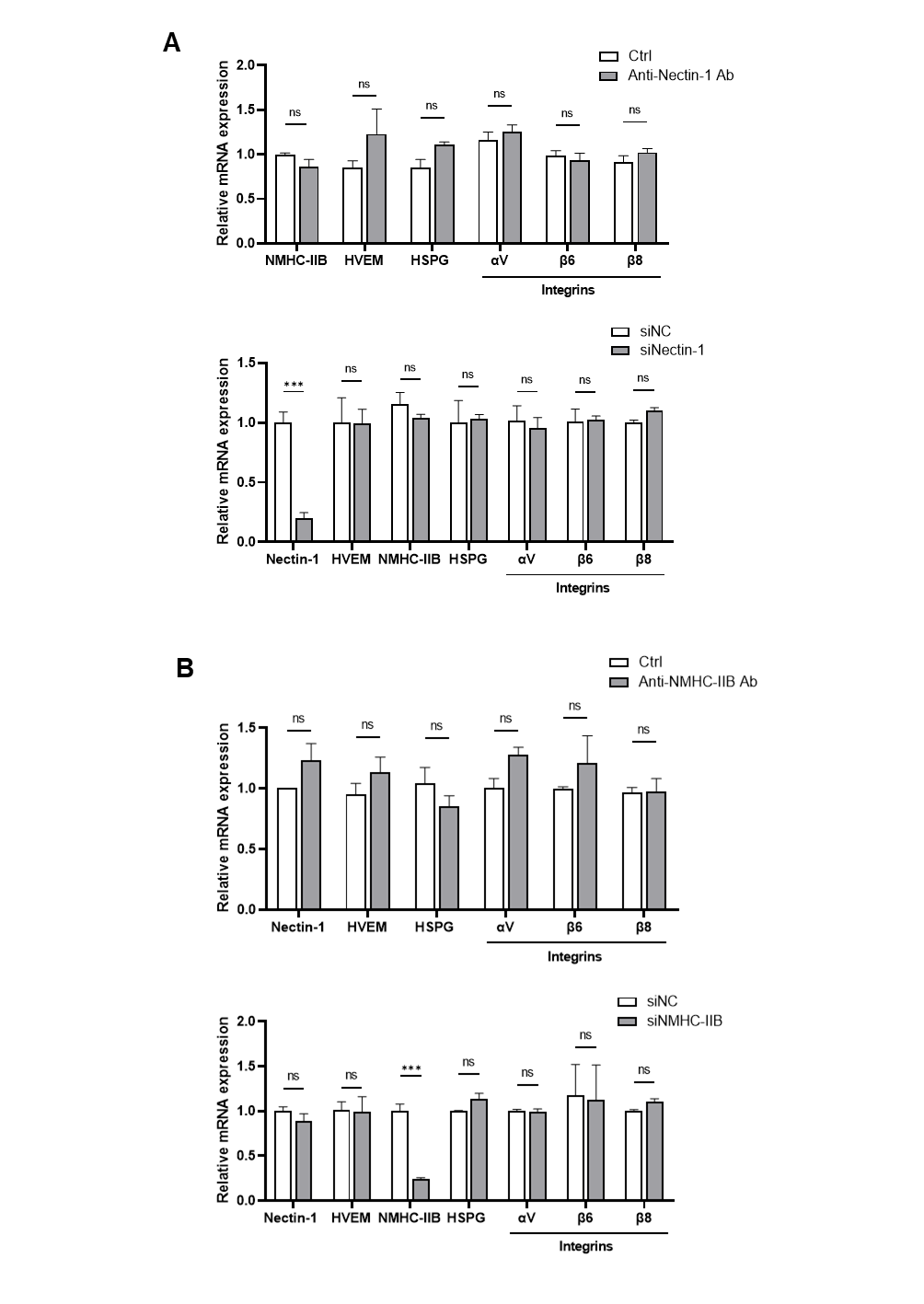

Supplement: Supplementary Figure 2 — Antibodies and siRNA of nectin-1 and NMHC-IIB did not affect other receptors involved in HSV-1 entry. (A) Cultured TG neurons were treated with antibody/siRNA of nectin-1 (n = 3). Cultured TG neurons with antibody diluent or NC siRNA were used as Ctrl or siNC. qPCR analysis showed that gD/gB receptors except nectin-1, HSPG, αvβ6- and αvβ8-integrins mRNA levels were no changes in the antibody/siRNA treatment groups compared to Ctrl or siNC. (B) Cultured TG neurons were treated with antibody/siRNA of NMHC-IIB (n = 3). Cultured TG neurons with antibody diluent or NC siRNA were used as Ctrl or siNC. qPCR analysis showed that gD/gB receptors except NMHC-IIB, HSPG, αvβ6- and αvβ8-integrins mRNA levels were no changes in the antibody/siRNA treatment groups compared to Ctrl or siNC. Bars denote SD. ns: not significant, ***P < 0.001 vs. Ctrl or siNC. [file Image_2.TIF]

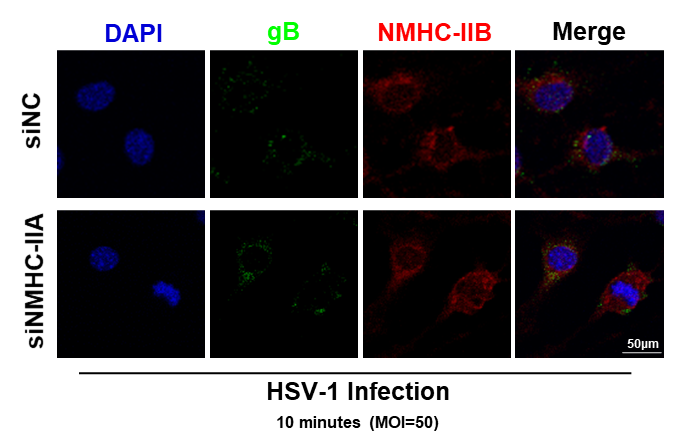

Supplement: Supplementary Figure 3 — No difference in the localization of NMHC-IIB after HSV-1 exposure when NMHC-IIA was knocked down. Cultured TG neurons were exposed to HSV-1 strain McKrae at an MOI of 50 at 4°C for 1 h, followed by a temperature shift to 37°C for 10 min. Shown is immunofluorescence analysis of NMHC-IIB and gB expression and localization. There was no difference in the localization of NMHC-IIB after HSV-1 exposure when NMHC-IIA was knocked down. [file Image_3.TIF]
