# Supplementary material for: Nectin-1 and Non-muscle Myosin Heavy Chain-IIB: Major Mediators of Herpes Simplex Virus-1 Entry Into Corneal Nerves
Source: Front Microbiol. 2022 Feb 28;13:830699. doi: 10.3389/fmicb.2022.830699 (PMC8919962; doi:10.3389/fmicb.2022.830699)
Supplement: Supplementary file 4 [file Table_1.DOCX]

# Table S1. Antibodies for blocking

| **Antibodies** | **Primer sequences** |
| --- | --- |
| **Nectin-1** | Abcam (ab66985) |
| **HVEM** | Abcam (ab47677) |
| **MAG** | Santa Cruz Biotechnology (sc-166849) |
| **NMHC-IIA** | Biolegend (PRB-440P) |
| **NMHC-IIB** | Biolegend (PRB-445P) |
