# Supplementary material for: Nectin-1 and Non-muscle Myosin Heavy Chain-IIB: Major Mediators of Herpes Simplex Virus-1 Entry Into Corneal Nerves
Source: Front Microbiol. 2022 Feb 28;13:830699. doi: 10.3389/fmicb.2022.830699 (PMC8919962; doi:10.3389/fmicb.2022.830699)
Supplement: Supplementary file 5 [file Table_2.DOCX]

# Table S2. Primer sequences of siRNAs

| **Gene** | **Primer sequences** |
| --- | --- |
| **siNectin-1** | AGGTGAACGACTCCATGTA |
| **siHVEM** | GCATTTCAACAGGAAGTAA |
| **siNMHC-IIA** | AGGCUGCAGACAAGUACCUCUTT |
| **siNMHC-IIB** | GCGUUUGGCUCUGCAUACAAA |
