# Supplementary material for: Nectin-1 and Non-muscle Myosin Heavy Chain-IIB: Major Mediators of Herpes Simplex Virus-1 Entry Into Corneal Nerves
Source: Front Microbiol. 2022 Feb 28;13:830699. doi: 10.3389/fmicb.2022.830699 (PMC8919962; doi:10.3389/fmicb.2022.830699)
Supplement: Supplementary file 6 [file Table_3.DOCX]

# Table S3. Primer sequences used in qPCR

| **Gene** | **Forward primers** | **Reverse primers** |
| --- | --- | --- |
| **Ms-GAPDH** | TGATGACATCAAGAAGGTGGTGAAG | TCCTTGGAGGCCATGTGGGCCAT |
| **HSV-1 ICP0** | ATGTCTGGGTGTTTTTCCCTGC | TCTCGAACAGTTCCGTGTCC |
| **Ms-Nectin-1** | GTCCTGGGAAACACGGCTAA | TAGGTCCCCGGAAGAAGAGG |
| **Ms-HVEM** | CCACTGTTCCACATGCTTGC | GCTGTTGGTCCCACGTCTTA |
| **Ms-3-OST-2** | GACGAGGACTGGACTGGTAC | GCACCACCACTATCAGCTTG |
| **Ms-MAG** | CTGCCGCTGTTTTGGATAATGA | CATCGGGGAAGTCGAAACGG |
| **Ms-NMHC-IIA** | GGCCCTGCTAGATGAGGAGT | CTTGGGCTTCTGGAACTTGG |
| **Ms-NMHC-IIB** | GGAATCCTTTGGAAATGCGAAGA | GCCCCAACAATATAGCCAGTTAC |
| **HSV-1 gB** | AACGCGACGCACATCAAG | CTGGTACGCGATCAGAAAGC |
| **Ms-HSPG** | TTCCAGATGGTCTATTTCCGGG | CTTGGCACTTGCATCCTCC |
| **Ms-Integrin subunit alpha V** | CGGGTCCCGAGGGAAGTTA | TGGATGAGCATTCACATTTGAGA |
| **Ms-Integrin subunit beta 6** | ATGGGGATTGAGCTGGTCTG | GACAGGTGGGTGAAATTCTCC |
| **Ms-Integrin subunit beta 8** | TGCATGTTGTAACGTCAAGTGA | GATGCTGACACATCAACCAGATA |
