# Supplementary material for: Nectin-1 and Non-muscle Myosin Heavy Chain-IIB: Major Mediators of Herpes Simplex Virus-1 Entry Into Corneal Nerves
Source: Front Microbiol. 2022 Feb 28;13:830699. doi: 10.3389/fmicb.2022.830699 (PMC8919962; doi:10.3389/fmicb.2022.830699)
Supplement: Supplementary file 7 [file Table_4.DOCX]

# Table S4. Antibodies for immunofluorescence and western blotting

| **Antibodies** | | **Company**  **(Catalogue number)** | **Dilution** |
| --- | --- | --- | --- |
| **Primary**  **Antibodies** | **GAPDH** | Bioss  (bsm-33033M) | 1:5000 (WB) |
|  | **HSV-1 ICP0** | Santa Cruz Biotechnology  (sc-53070) | 1:200 (IF) |
|  | **HSV-1 gD** | Santa Cruz Biotechnology  (sc-21719) | 1:200 (IF) |
|  | **HSV-1 gB** | Santa Cruz Biotechnology  (sc-56987) | 1:200 (IF) |
|  | **Nectin-1** | Abcam  (ab66985) | 1:1000 (WB)  1:200 (IF) |
|  | **HVEM** | Abcam  (ab47677) | 1:1000 (WB)  1:200 (IF) |
|  | **MAG** | Santa Cruz Biotechnology  (sc-166849) | 1:100 (IF) |
|  | **NMHC-IIA** | Biolegend  (PRB-440P) | 1:1000 (WB)  1:500 (IF) |
|  | **NMHC-IIB** | Biolegend  (PRB-445P) | 1:1000 (WB)  1:500 (IF) |
| **Secondary Antibodies** | **β III tubulin (NL557)** | R&D Systems  (NL1195R) | 1:10 (IF) |
|  | **HSV-1 (FITC)** | Abcam  (ab20437) | 1:20 (IF) |
|  | **Donkey Anti-Mouse IgG H&L (Alexa Fluor® 488)** | Abcam  (ab150105) | 1:1000 (ICC)  1:400 (IF) |
|  | **Donkey Anti-Rabbit IgG H&L (Alexa Fluor® 555)** | Abcam  (ab150074) | 1:1000 (ICC)  1:400 (IF) |
|  | **Donkey Anti-Rabbit IgG H&L (Alexa Fluor® 647)** | Abcam  (ab150075) | 1:1000 (ICC)  1:400 (IF) |
|  | **Goat Anti-Mouse IgG (HRP)** | Bioss  (bs-0296-HRP) | 1:5000 (WB) |
|  | **Goat Anti-Rabbit IgG (HRP)** | Biosharp  (BL003A) | 1:5000 (WB) |
